# Helium Irradiation and Implantation Effects on the Structure of Amorphous Silicon Oxycarbide

**DOI:** 10.1038/s41598-017-04247-x

**Published:** 2017-06-20

**Authors:** Qing Su, Shinsuke Inoue, Manabu Ishimaru, Jonathan Gigax, Tianyao Wang, Hepeng Ding, Michael J. Demkowicz, Lin Shao, Michael Nastasi

**Affiliations:** 1 Nebraska Center for Energy Sciences Research, University of Nebraska-Lincoln, Lincoln, NE 68583-0857 USA; 2 Department of Materials Science and Engineering, Kyushu Institute of Technology, Tobata, Kitakyushu, Fukuoka, 804-8550 Japan; 3 Department of Nuclear Engineering, Texas A&M University, College Station, TX 77843-3128 USA; 4 Materials Science and Engineering, Texas A&M University, College Station, TX 77843 USA; 5 Department of Mechanical and Materials Engineering, University of Nebraska-Lincoln, Lincoln, NE 68583-0857 USA; 6 Nebraska Center for Materials and Nanoscience, University of Nebraska-Lincoln, Lincoln, NE 68588-0298 USA

## Abstract

Despite recent interest in amorphous ceramics for a variety of nuclear applications, many details of their structure before and after irradiation/implantation remain unknown. Here we investigated the short-range order of amorphous silicon oxycarbide (SiOC) alloys by using the atomic pair-distribution function (PDF) obtained from electron diffraction. The PDF results show that the structure of SiOC alloys are nearly unchanged after both irradiation up to 30 dpa and He implantation up to 113 at%. TEM characterization shows no sign of crystallization, He bubble or void formation, or segregation in all irradiated samples. Irradiation results in a decreased number of Si-O bonds and an increased number of Si-C and C-O bonds. This study sheds light on the design of radiation-tolerant materials that do not experience helium swelling for advanced nuclear reactor applications.

## Introduction

The development of new, radiation tolerant materials is crucial to deploy the next generation fission reactors^1–5^. Different strategies have been explored to improve radiation tolerance of structural materials and suppress radiation induced dimensional and property changes. In particular, efforts have been made to introduce interfaces between nanoscale oxides particles and the metal matrix in oxide dispersion strengthened (ODS) steels as point-defect sinks to mitigate radiation damage and suppress swelling^6, 7^. Several incoherent interfaces in nanoscale metallic laminates, such as Cu/V^8^, Cu/Nb^9, 10^, and Fe/W^11^, have exhibited strong sink strength and suppressed He bubble formation. The grain boundaries in nanocrystalline metals, although facing challenges of grain stability under irradiation, have shown to assist defect annihilation^12, 13^.

An alternate method to manage point defect is to develop thermally stable amorphous materials that inherently do not exhibit point defects. Instead of generating vacancies and interstitials during irradiation, amorphous materials produce fluctuations in free volume or local bonding topology that can easily recover^14^. These materials may serve as the basis for developing a new class of radiation tolerant structural materials. Amorphous SiOC is a model material which consists of nanoscale structural units of SiO_x_C_4−x_ with x = 0, 1, 2, 3 or 4. The collection of these tetragonal nanoscale building blocks gives rise to high crystallization temperature and high radiation tolerance. Previous results have demonstrated that amorphous SiOC possesses good thermal and irradiation stability over a wide range of compositions, irradiation doses, and irradiation temperatures^15–20^. The material showed no evidence of crystallization up to a temperature of 1200 °C for an annealing time of 2.0 hours. In addition, amorphous SiOC films remained amorphous after both light ion (He) and heavy ion (Kr) irradiation within a wide envelope of irradiation conditions.

Although previous X-ray diffraction (XRD), high resolution transmission electron microscopy (TEM) and electron diffraction results suggest that SiOC alloys retain their amorphous state after a wide range of irradiation conditions^15, 16^, these techniques do not assess changes in atomic-level structure of this amorphous alloys before and after irradiation/helium implantation. Such information is crucial for understanding irradiation effects and for establishing structure-property relationships in irradiated amorphous materials. One approach for examining structural information of amorphous materials is to measure PDF. The PDF is a pair correlation that represents the probability of finding atoms as a function of radial distance, r, from an average center atom. Structural information such as the distribution of interatomic distances, bond angles, and coordination number is embedded in the PDF peak positions, widths, and relative intensities. PDF has been widely used to examine the short-to-medium range order in different metallic glasses and covalent amorphous solid^21–23^. Therefore, in the present work, we use electron elastic scattering to obtain the radial distribution function of amorphous SiOC film, aiming to probe the amorphous structure information before and after irradiation/implantation.

## Results

### Experimental design

**Figure 1:**
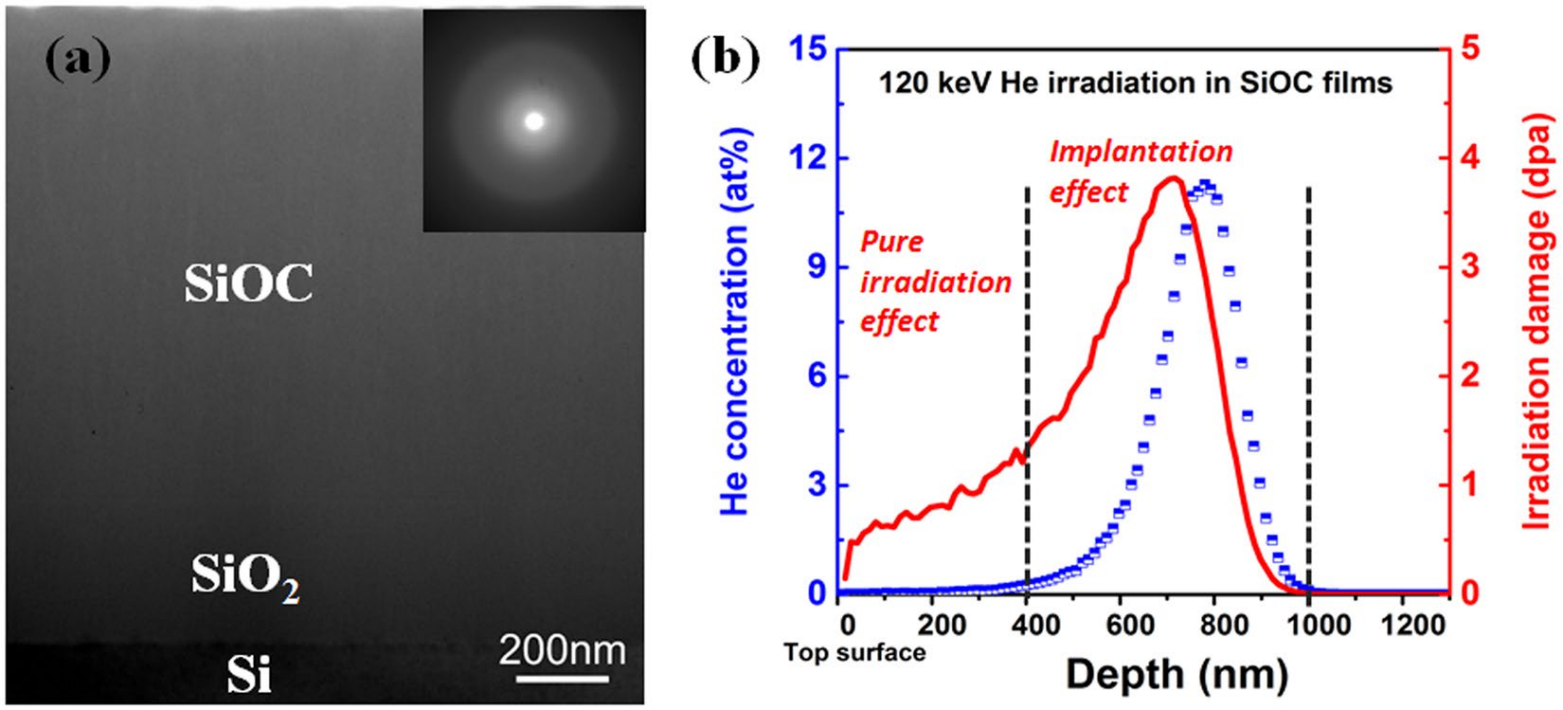
(**a**) Cross-sectional TEM image of as-deposited SiOC film. The inset is the corresponding selective area diffraction pattern. (**b**) Simulated depth profile of helium concentration and irradiation damage for SiOC films after 120 keV He irradiation.

In order to examine both irradiation and He implantation effect on the PDF of amorphous SiOC, 120 keV He ion irradiation was selected. Figure 1a presents the typical cross-sectional TEM image of as-deposited SiOC film. The thickness of the as-prepared film is approximately 1 micron. The selected area diffraction pattern (inset of Fig. 1a) shows no sign of long range order. The simulated depth profiles of implanted He concentration and He irradiation damage are shown in Fig. 1b which reveal the presence of two regions of interest. The first region is the top 400 nm area where almost no helium implantation occurs and the material mainly experiences irradiation damage; the second region is from 400 nm to 1 micron where both He implantation and irradiation damage occur simultaneously. In this work, the PDF at 200 and 800 nm from top surface were chosen to examine the structure of these two representative regions. For an average 1 dpa irradiation in the 200 nm region (0.04 at% He implantation), approximately 3 dpa of irradiation damage and a He implantation peak concentration of 11.3 at% are obtained at a depth of ~800 nm. Because the He irradiation damage and He implantation concentration in the film are proportional to the total He fluence, the damage and He depth profiles of other doses will scale accordingly.

### TEM and PDF characterization

**Figure 2:**
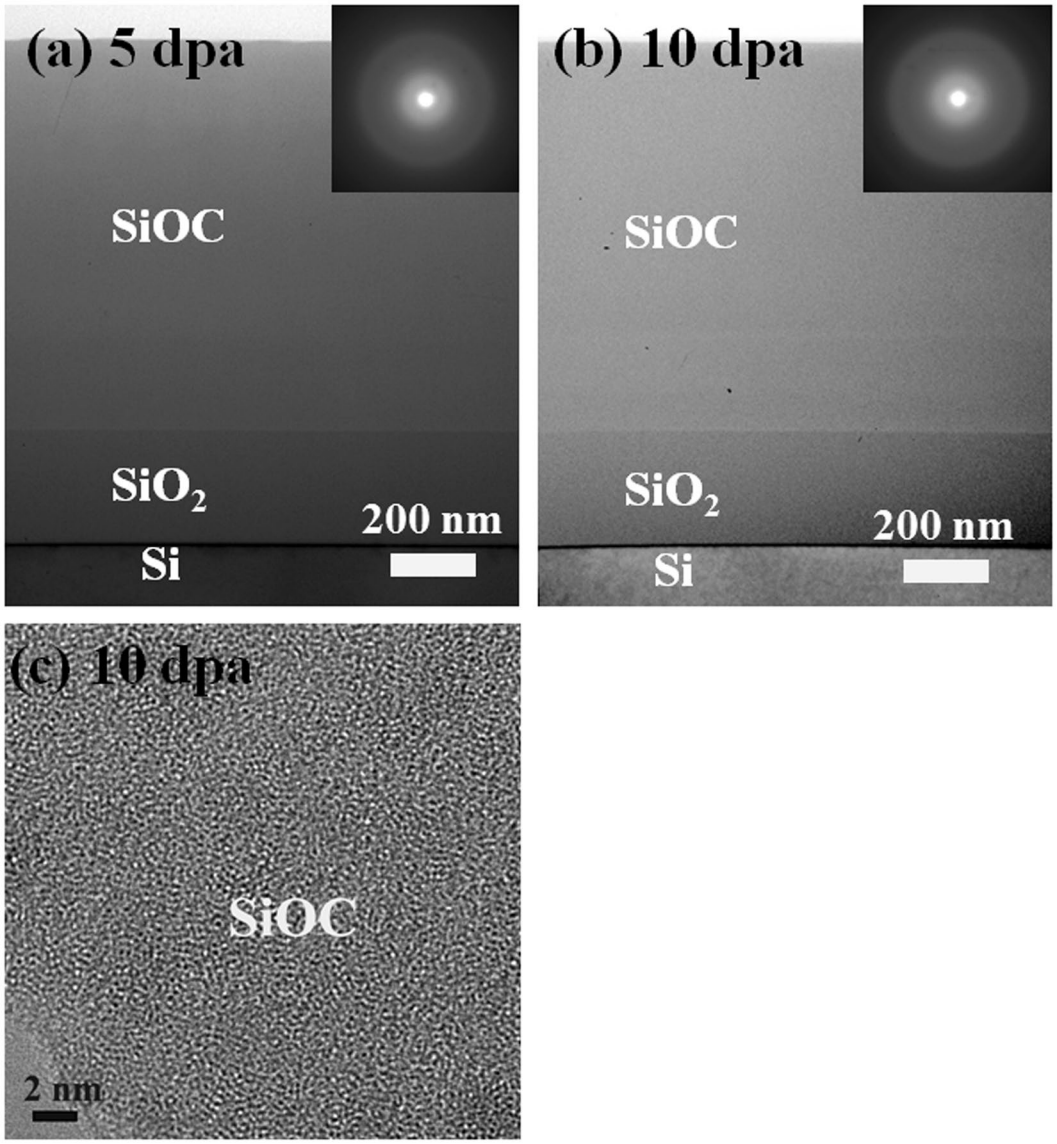
Cross-sectional TEM micrographs of SiOC film after (**a**) 5 (15) and (**b**) 10 (30) dpa irradiation at 200 nm (800 nm) regions. No void formation, element segregation or crystallization are present in pure irradiation region. (**c**) High resolution TEM image of SiOC film in the 20 nm region after 10 dpa irradiation exhibiting a maze-like pattern with no discernable structure.

Figure 2a and b show cross-sectional TEM micrographs of SiOC films after 5 and 10 dpa irradiation at 200 nm region, respectively. Both of micrographs exhibit uniform contrast throughout the whole film. No void formation, element segregation or crystallization are present in pure irradiation region (200 nm), up to the highest radiation damage level (10 dpa). More interestingly, no helium bubbles are observed at the simulated He peak position (800 nm) where the damage levels are 15 dpa (Fig. 2a) and 30 dpa (Fig. 2b). The thickness of SiOC film before and after He irradiation/implantation is approximately the same, ~1 micron. Under these conditions the SiOC alloy maintain its amorphous state with no helium bubble or void formation observed after 30 dpa (an equivalent applied dose if retained would lead to a 113 at% He implantation). It should be noted that non-Rutherford proton backscattering results showed that He was not retained in the SiOC to the maximum fluence applied (data not show here) and that rapid He diffusion in SiOC occurs^24^. These results are further confirmed by the corresponding selected area diffraction pattern in the inset of each figure, which shows diffuse halo rings. Figure 2c shows a high resolution TEM image of SiOC film after 10 dpa irradiation and exhibits a maze-like pattern with no discernable structure. These data clearly demonstrate the superior room temperature irradiation tolerance of the SiOC.

**Figure 3:**
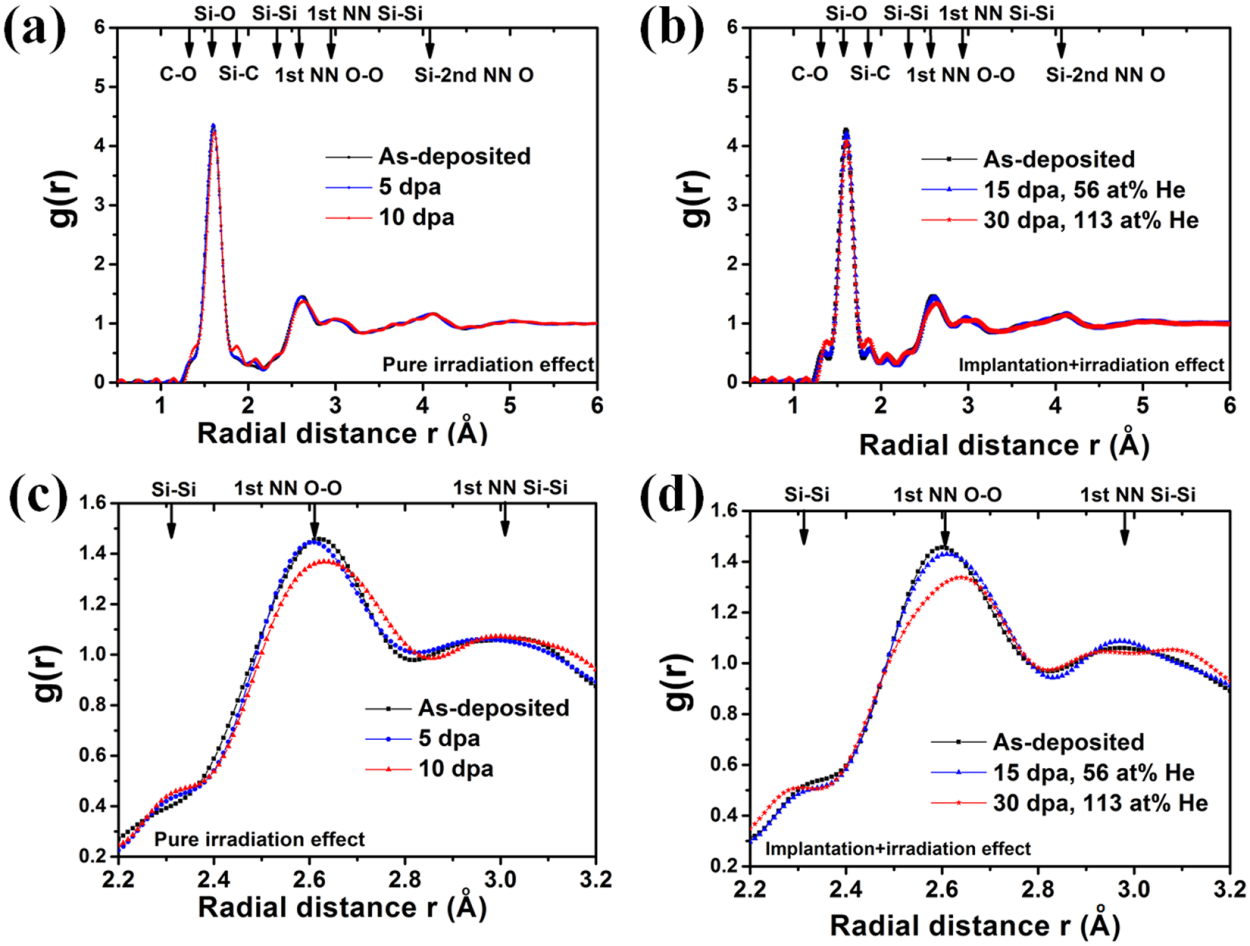
The PDF of SiOC film before and after (**a**) irradiation and (**b**) implantation. The magnified PDF of SiOC film before and after (**c**) irradiation and (**d**) implantation at the range from 2.2 to 3.2 Å.

In order to probe the structural information of amorphous SiOC, the electron diffraction patterns of SiOC samples before and after irradiation/He implantation are collected. The corresponding reduced PDF, g(r), of SiOC specimens before and after irradiation/He implantation are presented in Fig. 3a and b, respectively. The Fig. 3c and d show magnified PDF at the range from 2.2 to 3.2 Å. The bonding topology of the amorphous SiOC network is based on tetrahedral SiO_x_C_4−x_ units (X = 0, 1, 2, 3, 4). In the first shell, there are two main characteristic bond distances due to Si-O and Si-C bonds. The PDF reveals two peaks located at 1.60 Å and at 1.88–1.89 Å which correspond to Si-O and Si-C bonds, respectively^25, 26^. The features at 2.60, 2.99 and 4.10 Å are attributed to first nearest neighbor (NN) O-O, first NN Si-Si, and second NN Si-O pair distances. At larger radial distance over 5 Å, the PDF gradually converges to unity, indicating no long-range order/correlation exists in SiOC specimens before and after irradiation. For the as-deposited SiOC specimen, the value for the average bond angle O-Si-O (calculated from the Si-1st O and O-O distances) is approximately 109° which is in good agreement with a tetrahedral geometry. The calculated Si-O-Si bridging bond angle from the first NN Si-O and Si-Si distances is 139°. This value is slightly smaller than that observed for various forms of amorphous SiO_2_ which range from 144 to 151° in the literature^27–29^. The shoulder at 2.10 Å is due to the presence of SiO_x_C_4−x_ units (X = 1, 2, 3) or oxygen hole centers (≡Si-O•: an unpaired electron on an oxygen atom)^25^. The two small peaks at 1.35 and 2.32 Å are assigned to C-O and Si-Si bonds, respectively. The bond types, average interatomic distances and bond angles obtained for SiOC before and after irradiation/He implantation are summarized in Table 1. All of the findings confirm that the regular topology for the SiO_x_C_4−x_ units (X = 0, 1, 2, 3, 4) are present in the SiOC films before and after irradiation. As shown in Fig. 3 and Table 1, the change of peak location is very small suggesting very few structural variations or changes in atomic density before and after irradiation/He implantation.

**Table 1:** Average peak position of neighbor shells and bond angles in SiOC before and after He irradiation/implantation.

SiOC samples	Peak position (Å), and its representation						Si-O-Si Bond angle (°)
	First shell				Second shell		
	C-O	Si-O	Si-C	Si-Si	1^st^ NN O-O	1^st^ NN Si-Si	
As-deposited	1.34	1.60	1.86	2.32	2.62	3.00	139.3
5 dpa	1.34	1.60	1.87	2.32	2.62	3.00	139.3
10 dpa	1.35	1.60	1.87	2.32	2.62	3.00	139.3
15 dpa + 56 at% He	1.34	1.60	1.86	2.30	2.62	2.99	137.3
30 dpa + 113 at% He	1.37	1.61	1.87	2.28	2.63	2.99	135.5

### Molecular dynamic simulation

**Figure 4:**
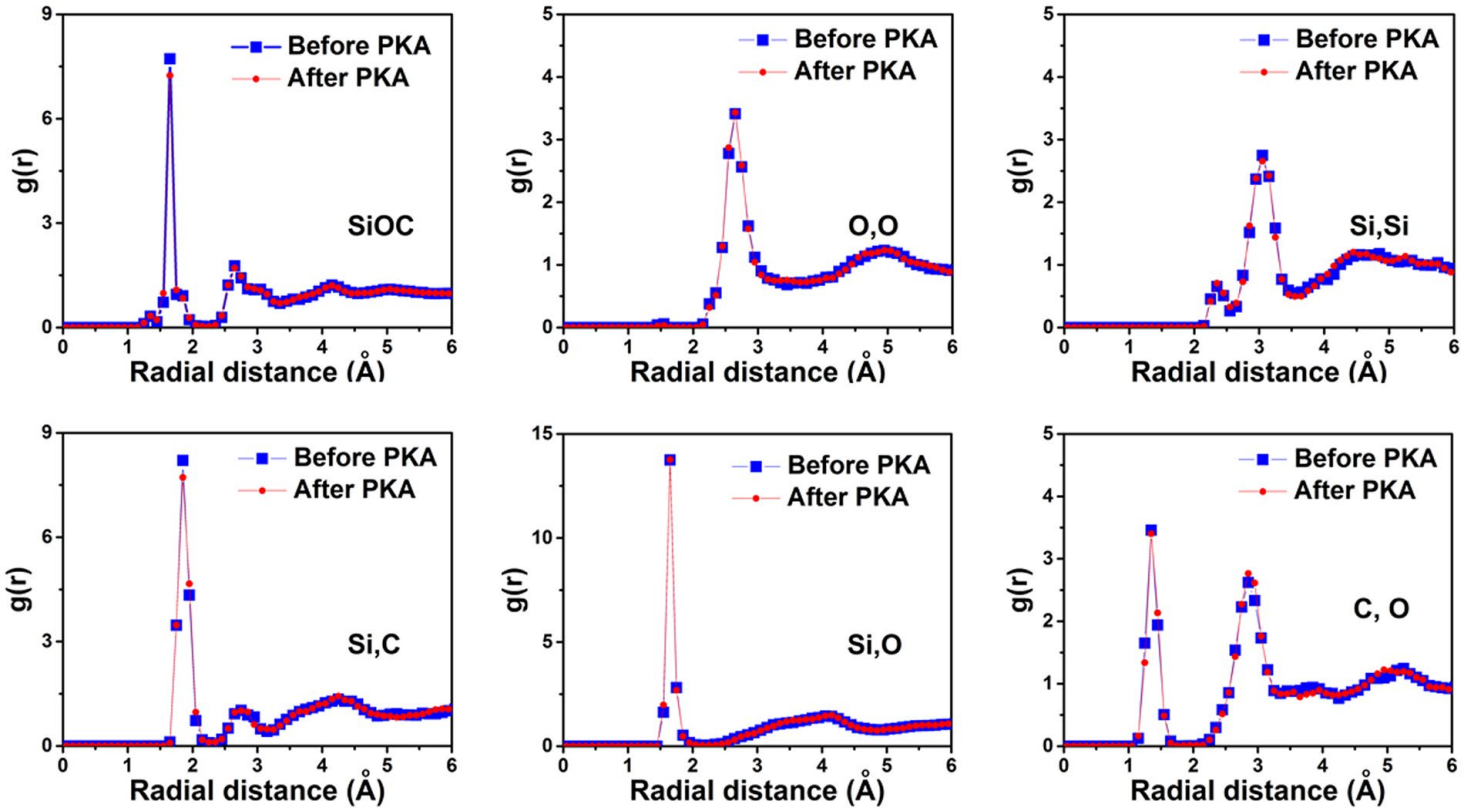
The first principles MD simulated PDF of SiOC before and after 0.1 dpa irradiation.

Some changes are observed for bond lengths and bond angles after irradiation, along with variations in several bond intensities, including Si-O, C-O, Si-Si and Si-C, indicating changes in the number of these bonds. As irradiation dose increases, the intensity of the C-O, Si-C peaks increases and the Si-O bond peak height decreases indicating that there are higher numbers of C-O, Si-C bonds and a lower number of Si-O bonds. As shown in Fig. 3c and d, the intensity of Si-Si bond peak gradually increases at low dpa level (<10 dpa) but decreases at high dpa level (>15 dpa), accompanying a peak shift. The peak height shift for the 1st NN O-O and 1st NN Si-Si is believed to be associated with changes of C-O, Si-O, Si-C, Si-Si bonds and structural relaxation after irradiation. The He implantation specimen exhibit similar PDF trends in that the number C-O, Si-C bonds increase and the number of Si-O bonds decreases. Figure 4 presents the PDF results of the SiOC film before and after ~0.1 dpa irradiation obtained by first principles molecular dynamic (MD) simulation. The simulated PDF plot of amorphous SiOC is almost the same as experimental obtained data, confirming the validity of the continuous random network model of bonding topology of SiOC films. The details about peak position and its representation are summarized in Table 2. The detailed structural variation as well as bonds formation and breaking at the atomic level in amorphous SiOC after irradiation can be found in ref. 30.

**Table 2:** MD simulated peak position and its representation.

Partial PDF	Peak position (Å), and its representation	
Si:O	1.67, Si-O bond	4.12, the 2^nd^ nearest neighbor (NN) O to Si distance
Si:C	1.86, Si-C bond	2.74, the 2^nd^ NN C to Si distance; 4.27, the 3^nd^ NN C to Si distance (similar to 2^nd^ NN O to Si)
Si:Si	2.35, Si-Si bond	3.06 (4.50), the 1^st^ (2^nd^) NN Si to Si distance
O:O	1.52, O-O bond	2.65 (4.98), the 1^st^ (2^nd^) NN O to O distance
C:O	1.36, C-O bond	2.87 (5.10), the 2^nd^ (3^rd^) NN C to O distance (similar to 1^st^ (2^nd^) NN O to O)

## Discussion

Through analysis of TEM and PDF measurements on amorphous SiOC films, we make several observations:

1. He irradiation leads to net Si-O bond breaking and net C-O, Si-C bond formation.
2. There is no void formation, elemental segregation, or recrystallization for SiOC after He irradiation and implantation. The material retains its amorphous structure.
3. No helium bubbles were observed and no volume swelling took place after even 130 at% helium implantation and 30 dpa. The helium implantation/irradiation results in few detectable structure changes in amorphous SiOC.

In this study, 120 keV He can initiate maximum 52.5 keV Si, 76.8 keV O and 90 keV C primary knock-on atoms (PKA). These PKA energy are much larger than the threshold displacement energy for Si, O, C elements (20–40 eV)^31, 32^, giving rise to ballistic displacements of atoms within materials. It is generally known that nuclear stopping processes can lead to net bond breaking in irradiated materials. For example, 10 dpa irradiation indicates that all atoms, on average, have been displaced 10 times suggesting significant Si-O, Si-C, Si-Si bonds breaking. Unlike experimental observations in irradiated amorphous SiO_2_ (a-SiO_2_), where Si nanocrystalline clusters are formed^33, 34^, no elemental segregation was observed although similar atomic displacement events took place in SiOC matrix. The results suggest that the introduction of C into amorphous SiO_2_ dramatically changes the materials’ response under irradiation. The introduction of C into amorphous SiO_2_ system changes the kinetics of structural relaxation in this material. Although similar amount of under- and over-coordinated Si and O defects can be found in SiO_2_ and SiOC, there is less possibility for cooperative movement of Si atoms in SiOC compared to that in the SiO_2_ matrix to form silicon cluster.

Nuclear stopping of Si, O, and C PKAs is sufficient to produce collision cascades and possibly thermal spikes, especially at the PKA’s end of range. Under these conditions the local temperature rises rapidly for several picoseconds then quenches to ambient temperature. Both ballistic displacements and thermal spikes give rise to irradiation damage in crystalline solids. However, in amorphous materials collision cascades may induce net bond formations and allow the amorphous material to undergo high-temperature structural relaxation^35^. It is expected that amorphous SiOC will go through significant structural relaxation after a 30 dpa irradiation. Interestingly, the structural relaxation of irradiated amorphous SiOC matrix seems to transform it from the as-deposited sputter-quenching metastable state to a more relaxed state. This relaxation process is associated with free volume and structure rearrangements. It is reported that the SiO_4_ tetrahedral unit that makes up a continuous random network can easily tilt, accommodating local mechanical strain in SiO_2_^36^. A similar response is expected in SiOC during the irradiation process. Interestingly, Shojaee *et al*. reported that sol-gel synthesized SiOC films evolved into a steady-state composition after heavy-ion irradiation which is quite closed to the composition of the SiOC film in this work which is Si-30 at%, O-40 at%, C-30 at%.

Compared to the region of the film which experienced just irradiation, the PDF for the deeper region which experienced both irradiation plus implantation showed no further change of the SiOC amorphous structure. These findings indicate that the amorphous SiOC structure reaches an energetic favorable state. He atomic diffusion appears to play a minimum role in changing the amorphous structure. Therefore, it is our hypothesis that the He atoms diffuse through the free volume of SiOC amorphous structure, exhibiting an interstitial-like diffusion mechanism. Future work is warranted to explore this idea.

## Conclusion

The structural information of amorphous SiOC before and after He irradiation/implantation has been examined by PDF study. He irradiation leads to Si-O net bonds breaking and C-O, Si-C net bonds formation. However, the material retained its glassy properties and no void formation, elemental segregation or recrystallization for SiOC was observed after 10 dpa irradiation. No helium bubbles were observed and no volume swelling took place even after an effective 113 at% helium implantation plus 30 dpa. The observations suggest that atomic displacing processes and helium implantation do not lead to SiOC degradation. This study, for the first time, provides structural information about amorphous SiOC after He irradiation/implantation. The understanding of irradiation/implantation effects on amorphous materials benefits the design of radiation-tolerant and helium-swelling resist materials for potential nuclear energy applications.

## Methods

### Specimen preparation

Amorphous SiOC alloys with composition of Si-30%, O-40%, C-30%; were synthesized via radio frequency (RF) co-sputtering from SiO_2_ and SiC targets at room temperature. Both the SiO_2_ (purity 99.995%) and the SiC (purity 99.5%) targets were obtained from AJA International, Inc. All of the SiOC films were deposited on surface oxidize Si substrates with a 300 nm top SiO_2_ layer. The thickness of SiOC film was approximate 1 micron. Prior to the sputtering deposition, the base pressure was 9.8 × 10^−6^ Pa or lower. The Ar partial pressure for SiO_2_ and SiC deposition was 0.65 Pa.

### Helium irradiation and PDF characterization

The SiOC films were subjected to 120 keV He ions irradiation at room temperature. The total fluences of 9.5 × 10^17^ and 1.9 × 10^18^ ions/cm^2^ were used to obtain averaged 5 and 10 dpa of damage at the pure irradiation region, respectively and 15 and 30 dpa in the irradiation plus implantation region, respectively. The depth-dependent damage and He concentration profiles were simulated by using the Stopping and Range of Ions in Matter (SRIM)-2008 software with the ion distribution and quick calculation of damage option^32^. A JEOL JEM-3000F Transmission Electron Microscope (TEM) with 300 kV operation voltage was employed to obtain high resolution images and electron diffraction patterns of SiOC thin films before and after irradiation. For atomic pair-distribution analysis, the samples were cooled by liquid nitrogen holder.

The cross-sectional TEM samples of SiOC films before and after irradiation were prepared by mechanical polishing and ion milling with a low glancing angle (5 degree). The acceleration voltage for Ar ion milling first adopted 4 keV and decreased to 1 keV at final stage (GATAN, PIPS) in order to minimize ion milling damage. The details about collecting electron diffraction pattern for atomic pair-distribution analysis have been described in previous works^23, 37^.

### Molecular dynamic simulation

The first principles molecular dynamics calculations were performed in SiOC, as described in ref. 30 Supercells containing 864 atoms with 18.75% C doping (21 Å × 21 Å × 28 Å) were simulated using VASP^38^, a plane wave based first-principles DFT code. We employed the Perdew-Burke-Ernzerhof (PBE)^39^ exchange-correlation functional within projector-augmented-wave approach^40^, a gamma-point only K point mesh, a 550 eV plane wave kinetic energy cutoff, and an energy convergence threshold of 10^−4^ eV for electronic self-consistent loop. Hard pseudopotentials of oxygen, carbon, and hydrogen, as well as standard pseudopotential for Si were used (O_h, C_h, H_h, and Si in VASP’s nomenclature, respectively).

To study material response under ion irradiation, a 100 eV primary knock-on atom (PKA) is introduced to simulate the unit displacement process. The PKA was initiated at a randomly selected Si atom because Si has large elastic scattering cross section and is therefore likeliest to suffer a collision with an incoming ion or neutron. A 0.25 femtosecond (fs) time step and NVE ensemble were used for PKA thermal equilibration process with duration of 0.375 picosecond (ps). A 1 fs time step and NVT ensemble with Langevin thermostat were used for annealing^41^, for which the process was performed in 100 K increments or decrements with a 0.5 ps equilibration after each temperature increment or decrement. Radial distribution functions (PDFs) at 300 K before PKA and after PKA are obtained.

We acknowledge that our simulation has been performed using a PKA with a 100 eV kinetic energy, so that it cannot represent a full-scale collision cascade. However, our simulation is representative of the mechanisms responsible for radiation damage in experiments that use much higher PKA energies, because the PKA initiates a localized, unit displacement process, which is responsible for most displacement-induced damage, according to the Kinchin-Pease (K-P) model^40^. Under K-P model, when a collision of an incident neutron with an atom transfers a kinetic energy larger than the displacement threshold, the atom displaces permanently from its original location, becoming a PKA. If this PKA has sufficiently high kinetic energy, it travels through the material producing higher-order knock-ons. However, if the PKA energy is close enough to the displacement threshold, it rapidly comes to rest, dissipating its remaining energy in a matter of picoseconds and generating a localized zone of dense structural damage. Consequently, most radiation-induced damage is created by knock-on atoms with relatively low energies close to the displacement threshold, which is the “unit displacement process” that we modeled here. Additional details about our modeling work may be found in ref. 30.
